# Backed Against a Wall: Iatrogenic Type A Aortic Dissection Pinned by Transcatheter Aortic Valve

**DOI:** 10.1016/j.shj.2023.100218

**Published:** 2023-09-15

**Authors:** Alexander E. Sullivan, Melissa M. Levack, Colin M. Barker, Kashish Goel

**Affiliations:** 1 Division of Cardiology, Vanderbilt University Medical Center, Nashville, Tennessee, USA; 2 Department of Cardiac Surgery, Vanderbilt University Medical Center, Nashville, Tennessee, USA

**Keywords:** Aortic dissection, Bicuspid aortic stenosis, Medical management, TAVR

A 72-year-old female with severe symptomatic aortic stenosis was referred for aortic valve replacement. She was found to have a bicuspid aortic valve with left ventricular outflow tract calcification (Figure 1a and b) and an enlarged ascending aorta (40 mm). She was considered prohibitive risk for surgery after evaluation by several surgeons due to multiple comorbidities and significant frailty that left her wheelchair bound. A transcatheter aortic valve replacement (TAVR) with Evolut Pro was planned.

**Figure 1:**
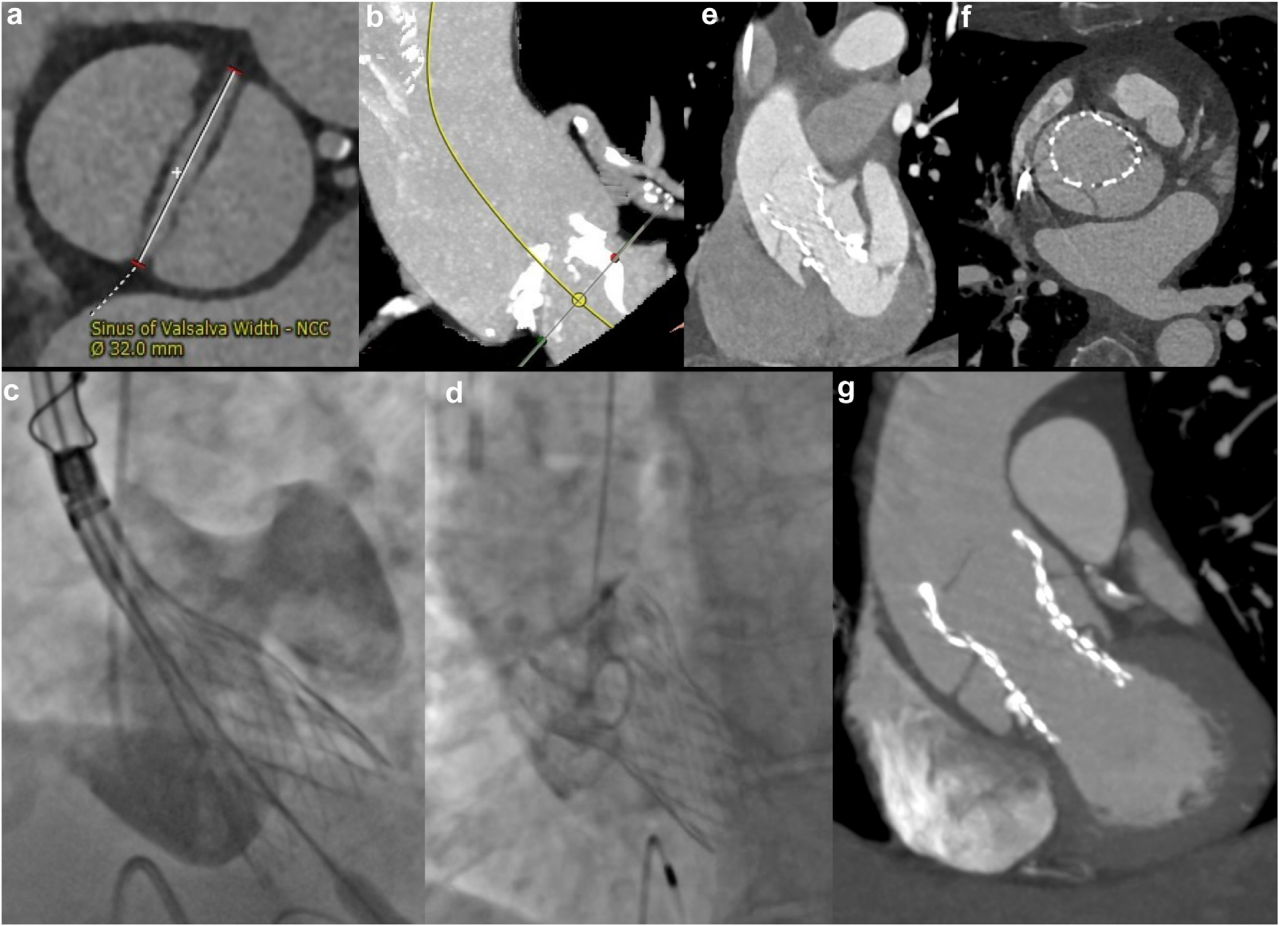
**Iatrogenic aortic root dissection.** (a) Cardiac CT: bicuspid aortic valve and (b) Heavy calcifications within aortic annulus and LVOT; (c) Aortogram: sinotubular aortic dissection during TAVR deployment; (d) Postdeployment aortogram: dissection pinned by TAVR cage; (e and f) Postprocedure CTA: sinotubular dissection; (g) 6-week follow-up CTA: stable dissection flap without extension. Abbreviations: CT, computed tomography; LVOT, left ventricular outflow tract; TAVR, transcatheter aortic valve replacement.

Intraprocedurally, there was significant difficulty advancing the Evolut valve across the native aortic valve due to aortic enlargement and angulation, which caused the body of the valve frame to deflect against the greater curve of the aorta and directed the nosecone into the coronary sinus (Figure 2). The valve was removed and balloon valvuloplasty was performed with a 20 mm True balloon. Despite this, the body of the valve continued to buck against the greater curvature. The nosecone repeatedly became lodged in the sinuses and multiple attempts to cross the aortic valve were unsuccessful. A 25 mm Gooseneck snare was then used to atraumatically pull the Evolut valve toward the inner curve of the ascending aorta, allowing passage across the annulus. The valve was deployed and then postdilated with a 24 mm True balloon.

**Figure 2:**
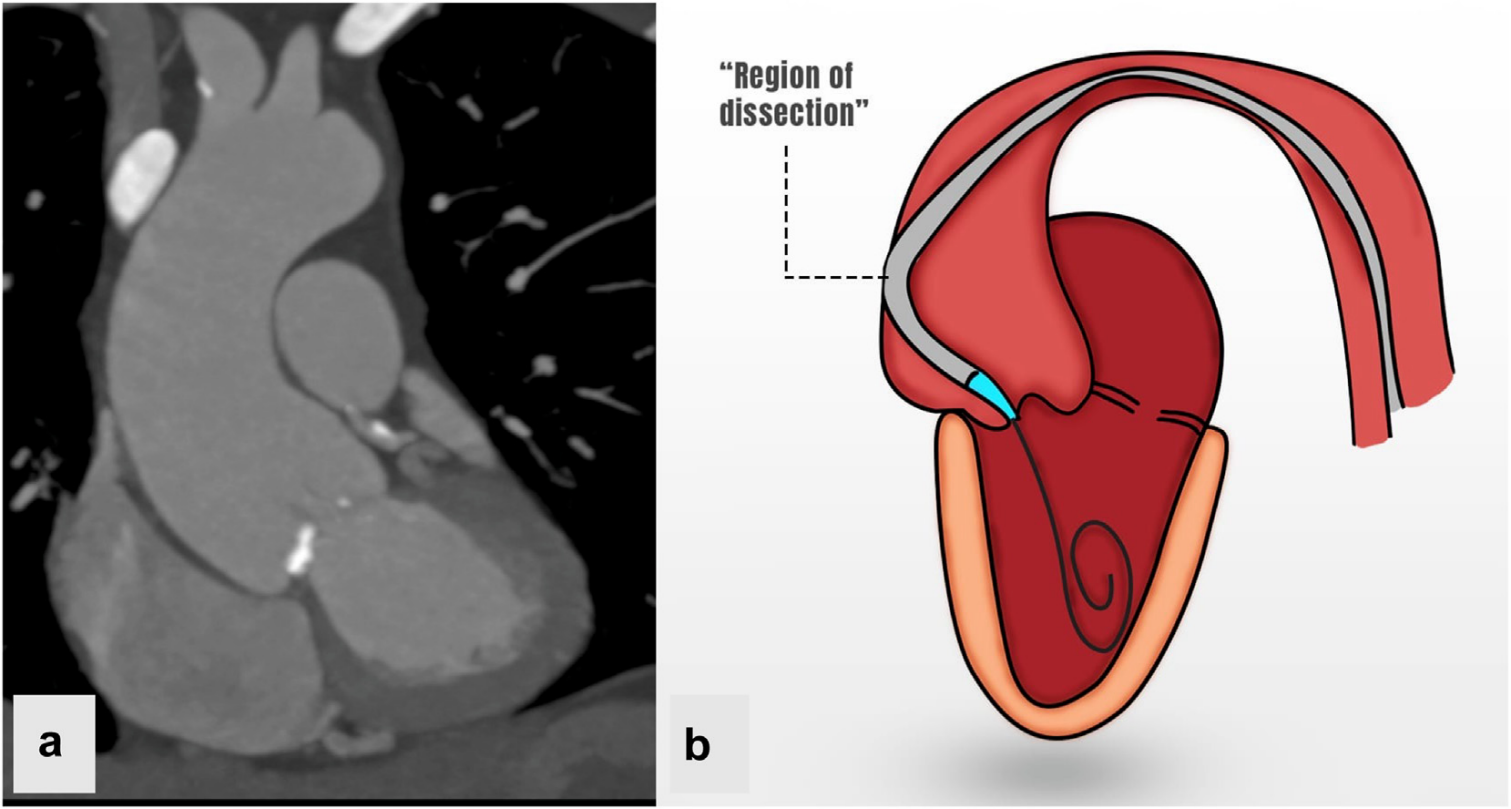
**Schematic drawing of the most likely mechanism of aortic injury.** Preprocedural computed tomography (a) showed an enlarged and angulated aorta. Equipment traversed the aortic valve at an acute angle deflecting against the greater curvature. Trauma to the greater curvature by the valve delivery system likely initiated dissection (b).

The patient reported chest pain 2 to 3 ​hours after the procedure, prompting review of the intraprocedural aortograms, which raised suspicion for an aortic root dissection (Figure 1c and d, Supplemental Videos 1 and 2). Computed tomography angiography (CTA) demonstrated a dissection beginning in the aortic root and sparing the coronary ostium. The flap appeared to be pinned behind the anterior aspect of the Evolut valve cage (Figure 1e and f, Supplemental Video 3). The patient was re-evaluated for open surgical repair, but again determined to have prohibitive risk. She was managed medically with esmolol titrated to a systolic blood pressure of 120 mmHg and heart rate of 60 bpm and demonstrated no signs of end-organ malperfusion or dissection propagation. She was discharged 72 ​hours later with return precautions.

At 6- and 12-week follow-up, the patient remained asymptomatic without dissection flap extension or propagation on CTA (Figure 1g). The external force provided by the self-expanding TAVR valve cage at the distal end of the dissection flap was thought to partially explain this stability. On 18-month post-TAVR CTA, the dissection flap was unchanged, and the patient remained asymptomatic.

Aortic dissection during TAVR is a rare but catastrophic complication occurring in ∼0.2 to 0.3% of TAVRs and presents a unique dilemma as many patients undergoing TAVR are at high or prohibitive surgical risk.^1^^,^^2^ It may happen secondary to intimal damage from stiff wires, device trauma, or balloon valvuloplasty injury. Iatrogenic type A dissection has traditionally been thought of, and managed, like spontaneous type A aortic dissection, which has a mortality rate exceeding 90% if left unrepaired. Heart team discussion and prompt surgical repair is the standard of care for those not at prohibitive surgical risk, but there is a growing body of evidence to support medical management with intravenous ß-blockade, dihydropyridine calcium channel blockers, or sodium nitroprusside to a systolic blood pressure of 120 mmHg and heart rate of 60 bpm.^3^^,^^4^ Other case reports have described successful management with endovascular thoracic aortic repair for those with prohibitive surgical risk who experience end-organ malperfusion despite medical therapy.^2^

The role of TAVR in patients with a bicuspid aortic valve and aortopathy is incompletely understood. Patients with bicuspid aortic valve have not been represented in TAVR trials, yet nearly 10% of all patients undergoing TAVR have a bicuspid aortic valve.^5^ Early case series suggested a higher rate of periprocedural complications and mortality in patients with bicuspid compared to tricuspid valves, but more contemporary cohorts suggest that this is not only feasible, but can be done safely.^6^ Patients with bicuspid valves will often have coexisting aortopathies, but only 25% will be large enough to necessitate concurrent prophylactic repair at the time of valve replacement.^5^ TAVR does not address the underlying aortopathy or any genetic predisposition to progressive dilation, but is a viable option in those without a surgical indication, as correcting underlying mechanical hemodynamic disturbances may slow progression.^5^ However, patients with underlying aortopathy are likely at increased risk for complications such as iatrogenic dissection, even with standard equipment manipulation and aortic wall trauma, as was seen in this case.

We present a medically managed case of iatrogenic type A aortic root dissection during TAVR. The dissection flap was fortuitously pinned behind the long Evolut frame, which maintained coronary perfusion, and a normally functioning TAVR valve maintained normal cardiac output through the left ventricular outflow track. This case adds to the growing body of evidence that iatrogenic type A aortic dissection can be successfully managed with medical therapy for impulse control in hemodynamically stable patients. Endovascular interventions are a potential bailout in those who ultimately require repair but are prohibitive surgical risk.

## Consent Statement

Patient consent was given for case publication.

## Funding

Alexander E. Sullivan is supported by the 10.13039/100000057National Institute of General Medical Science of the 10.13039/100000002National Institutes of Health under award number T32 GM007569.

## Disclosure Statement

Dr Goel receives intuitional research funding from Medtronic and serves as a proctor/consultant for Edwards LifeSciences and Abbott. Dr Barker receives intuitional research funding from Abbott, Medtronic, and Edwards LifeSciences. The other authors had no conflicts to declare.

## Supplementary Material

Supplemental Video 1 Aortogram during TAVR deployment showing dissection at the sinotubular junction.

Supplemental Video 2 Aortogram after TAVR deployment showing TAVR cage overlying dissection flap.

Supplemental Video 3 Cardiac computed tomography showing dissection at the sinotubular junction.
